# Silicon in the form of nanosilica mitigates P toxicity in scarlet eggplant

**DOI:** 10.1038/s41598-023-36412-w

**Published:** 2023-06-06

**Authors:** Deyvielen Maria Ramos Alves, Jairo Neves de Oliveira, Renato de Mello Prado, Patrícia Messias Ferreira

**Affiliations:** Department of Agricultural Production Sciences, Faculty of Agricultural and Veterinary Sciences, São Paulo State University (Unesp), Jaboticabal-SP. Access Prof. Paulo Donato Castellane s/n, Jaboticabal, SP 14884-900 Brazil

**Keywords:** Plant sciences, Plant stress responses

## Abstract

Intensive fertilization of vegetables can promote phosphorus (P) toxicity. However, it can be reversed using silicon (Si), although there is a lack of research clarifying its mechanisms of action. This research aims to study the damage caused by P toxicity to scarlet eggplant plants and whether Si can mitigate this toxicity. We evaluated the nutritional and physiological aspects of plants. Treatments were arranged in a 2 × 2 factorial design of two nutritional levels of adequate P (2 mmol L^−1^ of P) and toxic/excess P (8 to 13 mmol L^−1^ of P) combined with the absence or presence of nanosilica (2 mmol L^−1^ Si) in a nutrient solution. There were six replications. The excess P in the nutrient solution caused damage to scarlet eggplant growth due to nutritional losses and oxidative stress. We found that P toxicity can be mitigated by supplying Si, which decreases P uptake by 13%, improves C:N homeostasis, and increases iron (Fe), copper (Cu), and zinc (Zn) use efficiency by 21%, 10%, and 12%, respectively. At the same time, it decreases oxidative stress and electrolyte leakage by 18% and increases antioxidant compounds (phenols and ascorbic acid by 13% and 50%, respectively), and decreases photosynthetic efficiency and plant growth by 12% (by increasing 23% and 25% of shoot and root dry mass, respectively). These findings allow us to explain the different Si mechanisms used to reverse the damage caused by P toxicity to plants.

## Introduction

Scarlet eggplant (*Solanum aethiopicum*) is a vegetable of the Solanaceae family. It has a high nutritional value and a significant amount of antioxidant compounds^1^. It has economic importance for countries such as Brazil and some African countries^2^, such as Uganda, which cultivates this species mainly for commercialization because it is attractive in the local market due to its high nutritional value^3^. In Rwanda, it is the fifth most commercialized vegetable^4^. In Brazil, this species is important in the market because it is a typical vegetable in the its cuisine^5^. The States of Rio de Janeiro and Minas Gerais stand out in its cultivation, with an average productivity of 20 and 60 t ha^−1^^6^. However, the increase in growth and productivity of this species can be limited by different types of stress, especially abiotic ones, such as nutritional disorders^7^. Disturbance due to excess P may occur in some regions of intensive cultivation of vegetables when high doses of P are used either in hydroponic cultivation or in greenhouses with excess P fertilization^8^. Likewise, there are areas that use organic waste, such as compost sewage sludge, in cultivation^9^. During the decomposition process, waste releases organic acids that may block the same P adsorption sites in the soil solid phase, thus reducing the fixation of the P in the soil and greatly increasing the availability of P for plants^10^. In addition, there are soils in less weathered regions that, due to the predominance of a 2:1 clay mineral ratio and a lower percentage of Fe, aluminium (Al), and manganese (Mn) oxides, do not adsorb large amounts of P, facilitating its permanence in solution^11^. In both cases there will be an increase in P availability due to excessive dosage, consequently causing damage to plant metabolism^12^.

Despite this, P toxicity in plants may hasten or decrease the solubility of micronutrients in the root and decrease the content in leaves, impairing the functions of these nutrients in the metabolism, decreasing the production of biomass, and the use efficiency of these elements in the plant^13^. In addition, it may be associated with increased phytic acid synthesis in plants with excess P, as phytic acid has a high metal chelating activity with cytosol. The pH (around 7.5) is favourable to bound Pi, and divalent cations appear, such as Zn^2+^, Cu^2+^, and Fe^2+^^14^. Moreover, it may cause other types of damage, such as oxidative stress^14^, thus requiring further studies, especially on vegetables.

The problem of excess P in the soil is complex because under conditions of low pH (2 to 5), there is an increase in the adsorption of P, forming Fe and Al oxides. At the same time, there is a precipitation in the form of Fe phosphate and Al caused mainly due to the greater availability of these elements in the soil solution. On the other hand, under pH conditions of 6 to 10, P can also precipitate in the form of calcium phosphate, reducing its availability to plants^15^. However, P toxicity may occur in tropical soils that receive excess amounts of organic waste, such as sewage sludge and pig manure, as mentioned above. This can be explained by the occupation of P adsorption sites and the low mobility of this nutrient in the soil ^16^. Associated with this, soils that do not have significant leaching problems, such as very clayey ones, and low rainfalls can be a problem for plants when P is in excess in the soil solution^13^. Therefore, innovative strategies are important to allow the cultivation of vegetables and ensure an optimal growth in environments with excess P.

One option is the use of Si. It has been widely used in agriculture to mitigate many stresses, including disturbances of different nutrients ^17–22^. There is little information on P toxicity apart from a study focused on the vegetable *Cucumis sativus* ^23^. Most studies involving Si and stress mitigation were developed using non-nanometric sources. The advance in research on nanotechnology resulted in nanosilica^24^. Therefore, Si nanoparticles have been identified as an alternative source to make Si available to plants because of its size (less than 100 nm), which may increase Si absorption^25–29^ and improve the beneficial effects of these elements to plants. One of the known mechanisms of Si in mitigating different stresses is the improvement of the antioxidant defence system of plants, which may vary according to enzymatic and non-enzymatic mechanisms^30–36^. However, there is a lack of research specifically studying Si mechanisms involving the mitigation of P toxicity stress in scarlet eggplant plants.

In this scenario, it is pertinent to test the hypothesis that the supply of Si in the form of nanosilica, by promoting high absorption of Si by the plant, can reverse P toxicity in scarlet eggplant plants. It is not only a single mechanism, but also an association involving nutritional aspects, which favours better nutritional homeostasis, especially that of micronutrients. At the same time, it may induce synthesis of antioxidant compounds, strengthening the plant's antioxidant defence mechanisms.

To assess this hypothesis, this research aims to study the damage caused by P toxicity to scarlet eggplant plants and whether Si in the form of nanosilica allows mitigating this toxicity by evaluating the nutritional and physiological aspects of plants.

If the hypothesis is true, it will be possible to develop mechanisms of action of Si in mitigating P toxicity in scarlet eggplant plants, creating a sustainable strategy without risks of damage to the environment and allowing the cultivation of this vegetable in environments with excess P without losses in growth.

## Material and methods

### Plant material, growing conditions, and treatments

**Figure 1:**
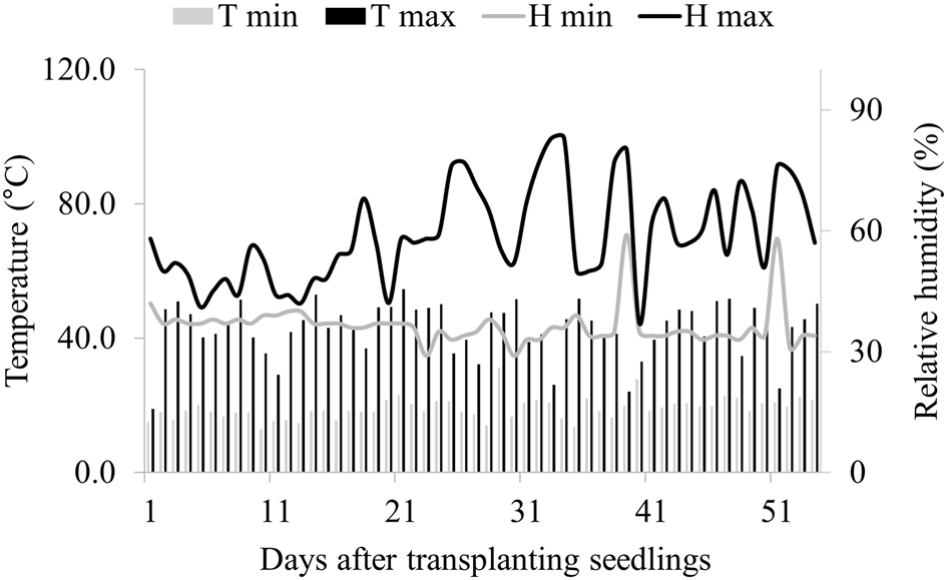
Data on meteorological variables, maximum (Tmax) and minimum (Tmin) air temperature and maximum (Hmax) and minimum (Hmin) relative air humidity during the experimental period.

The experiment was carried out in a closed hydroponic system with washed sand as substrate in a greenhouse with natural light and anti-aphid side screens. The monitoring of meteorological variables in the greenhouse was carried out using a thermo-hygrometer with daily recordings of temperature and relative air humidity for the whole experimental period. The average air temperature inside the greenhouse was 31 °C (Fig. 1).

Scarlet eggplant seedlings with four leaves were transplanted into polypropylene pots with a volume of 2 L. After transplanting, the seedlings received the nutrient solution of Hoagland and Arnon^37^. It is constituted by following macronutrients: nitrogen (N) (15 mmol L^−1^), P (1 mmol L^−1^), potassium (K) (6 mmol L^−1^), calcium (Ca) (4 mmol L^−1^), magnesium (Mg) (2 mmol L^−1^), and sulphur (S) (2 mmol L^−1^); and the following micronutrients: boron (B) (46 µmol L^−1^), Cu (0.3 µmol L^−1^), Fe (90 µmol L^−1^), Mn (12.6 µmol L^−1^), molybdenum (Mo) (0.1 µmol L^−1^), and Zn (1.3 µmol L^−1^). According to treatment, the solution was added starting at 10% of concentration and increasing it throughout the cultivation period. At 21 days, the concentration reached 90% and stabilized until the end of the experiment. Once a week, leaching was performed with deionized water to avoid excess salts.

The experiment had a 2 × 2 factorial design: nutrient solution with adequate P (1 mmol L^−1^) and excess P (8–13 mmol L^−1^) combined with a nutrient solution with absence of Si and presence of Si (2.0 mmol L^−1^). The experiment was completely randomized with six replications.

In the first application of the treatment with 10% concentration, the electrical conductivities (EC) were adequate P without Si (0.342 dS m^−1^), adequate P with Si (0.344 dS m^−1^), excess P without Si (0.342 dS m^−1^), and excess P with Si (0.344 dS m^−1^). As previously described, the concentration reached 90% and stabilized until the end of the experiment, as did the EC of treatments: adequate P without Si (3.102 dS m^−1^), adequate P with Si (3.104 dS m^−1^), excess P without Si, (3.230 dS m^−1^), and excess P with Si (3.232 dS m^−1^).

For treatments with Si, the nutrient solution was applied to the root using nanosilica Bindzil® (Si: 168.3 g L^−1^; specific surface area: 300 m^2^ g^−1^; pH: 10.5; density: 1.2 g cm^−3^; Na_2_O: 0.5%; viscosity: 7 centi Poise – cP). The Si concentration used followed the indication of 2 mmol L^−1^, which is adequate to prevent polymerization and promote the absorption of the element by plants^38^.

We started applying the treatments after transplanting red eggplants that had four leaves. The treatment with excess P initially used a solution with 8 mmol L^−1^ of P and, after fifteen days, it reached 13 mmol L^−1^ of P until the plant showed typical symptoms of P toxicity. This occurred with a great visual symptomatology at 52 days after transplanting seedlings. The plants were then in pre-flowering.

To prepare the nutrient solution, deionized water was used with a pH value close to 7.0. After the addition of salts and the respective treatments, the pH value was corrected to 5.5–6.0 with a solution of hydrochloric acid at 1 mmol L^−1^ and sodium hydroxide whenever necessary.

### Growth assessment

Evaluations of growth were performed 53 days after transplanting. The plants were in pre-flowering and showed characteristic symptoms of excess P. Using a ruler, plant height was measured, a digital calliper measured stem diameter, the leaves were counted, and an integrator (L3100, LiCor, USA) was used to quantify leaf areas.

### Dry mass production

53 days after transplanting, the plants were at the beginning of the reproductive phase (first emission of floral buds) and presented a characteristic symptomatology of excess P. The plants were then collected and washed in three solutions, starting with deionized water, then a detergent solution (0.1%), and finally a hydrochloric acid solution (0.3%). At the end, only deionized water was used^13^. Then, the plant material was dried in an oven with forced air circulation (± 65 °C) until constant mass and weighed on a precision analytical scale. The values of shoot dry mass (leaves and stem) and root dry mass were calculated.

### Contents, accumulation, and use efficiency of nutrients and Si

The contents of the macronutrients P and N and of the micronutrients Cu, Fe, and Zn were analysed in shoots using the method described by^39^. The accumulation of nutrients was determined following the methodology proposed by^40^. The results were expressed as content per dry mass of shoots (g per plant). The use efficiency of micronutrients (Cu, Fe, and Zn) was calculated by the ratio between shoot dry mass squared by the accumulation of micronutrients in shoots^41^. Nutrient accumulation (in mg per plant) was calculated by the product of nutrient content (g kg^−1^) and shoot dry mass (g per plant) divided by 1,000. To determine the carbon content in plants, wet digestion of dry matter was performed with a K_2_Cr_2_O_7_ solution and external heat according to the methodology adapted from Walkley–Black presented by^42^. The Si content was determined by extracting the element according to the method described by^43^. Reading was taken by a spectrophotometer at an absorbance of 410 nm^44^.

### Pictures of plants

At 52 days after transplanting, the plants showed evident symptoms typical of P toxicity and were photographed for visual diagnosis.

### Total phenols

To determine total phenols, 0.1 g of living tissue was collected from the fully developed leaf. This sample was placed in a test tube to which 2 mL of methanol were added. The samples remained for three hours at room temperature at an average of 25 °C. After three hours, 3 mL of methanol were added, totalling 5 mL. 1 mL of this aliquot was removed, transferred to another test tube, also covered with Al foil, containing 10 mL of deionized water, and 0.5 mL of Folin-Ciocalteu (2N). Then, the tubes rested again at room temperature with the absence of light for three minutes. After resting, 1.5 mL of 20% sodium carbonate was added and kept for two hours in an environment with no light at room temperature. Then, sample readings were taken using a spectrophotometer at an absorbance of 765 nm. The levels obtained were calculated as gallic acid-equivalent (GAE) and expressed in g GAE 100 g^−1^ following the methodology described by^45^.

### Analysis of electrolyte leakage

The electrolyte leakage index was obtained from samples of five leaf discs of known area placed in beakers with 20 mL of deionized water. The difference between the initial and final electrical conductivity of the solution was multiplied by 100, as described by ^46^.

### Photosystem II quantum efficiency (Fv/Fm)

Using a portable fluorometer (Os30P + , Opti-Sciences Inc., USA), measurements of the quantum efficiency of the photosystem II (Fv/Fm) were made on the fully developed leaf^47^.

### Ascorbic acid determination

The determination of ascorbic acid in plant samples with the collection of 0.100 g of living tissue from fully developed leaves was performed using the 2,6-dichlorophenol-indophenol method, as described by^48^. The contents were calculated as proportional ratios based on the standard titration of ascorbic acid and expressed as mg kg^−1^ per 100 g of dry mass.

### Quantification of chlorophyll and carotenoids

The concentrations of chlorophyll a and b and carotenoids were determined by collecting discs from leaves of living tissues with a mass of 0.025 g to 0.030 g, added to an Eppendorf tube with 1.5 ml of 80% acetone, and stored in a dark place or under light. After the complete depigmentation of disks, a spectrophotometer reading was taken at an absorbance of 663 nm for chlorophyll a, 647 nm for chlorophyll b, and 470 nm for carotenoids^49^.

### Analysis of results

Data normality was verified using the Shapiro–Wilk test and homoscedasticity using the Levene test. The data obtained were subjected to 5% probability analysis of variance. When the F test was significant, the means were subjected to a factor analysis to test the effects of different P (adequate and excessive) conditions and interactions (P x Si). The means were compared using the Tukey test at a significance level of 5% (*p* < 0.05). All analyses were performed using the R software, version 4.1.0. 37^50^. To plot the graphical representation of the results, we used the means and the mean standard error of the variables evaluated.

### Research involving plants

The experimental research was carried out in accordance with the relevant institutional, national, and international guidelines and legislation and, still, does not involve an endangered species.

## Results

**Figure 2:**
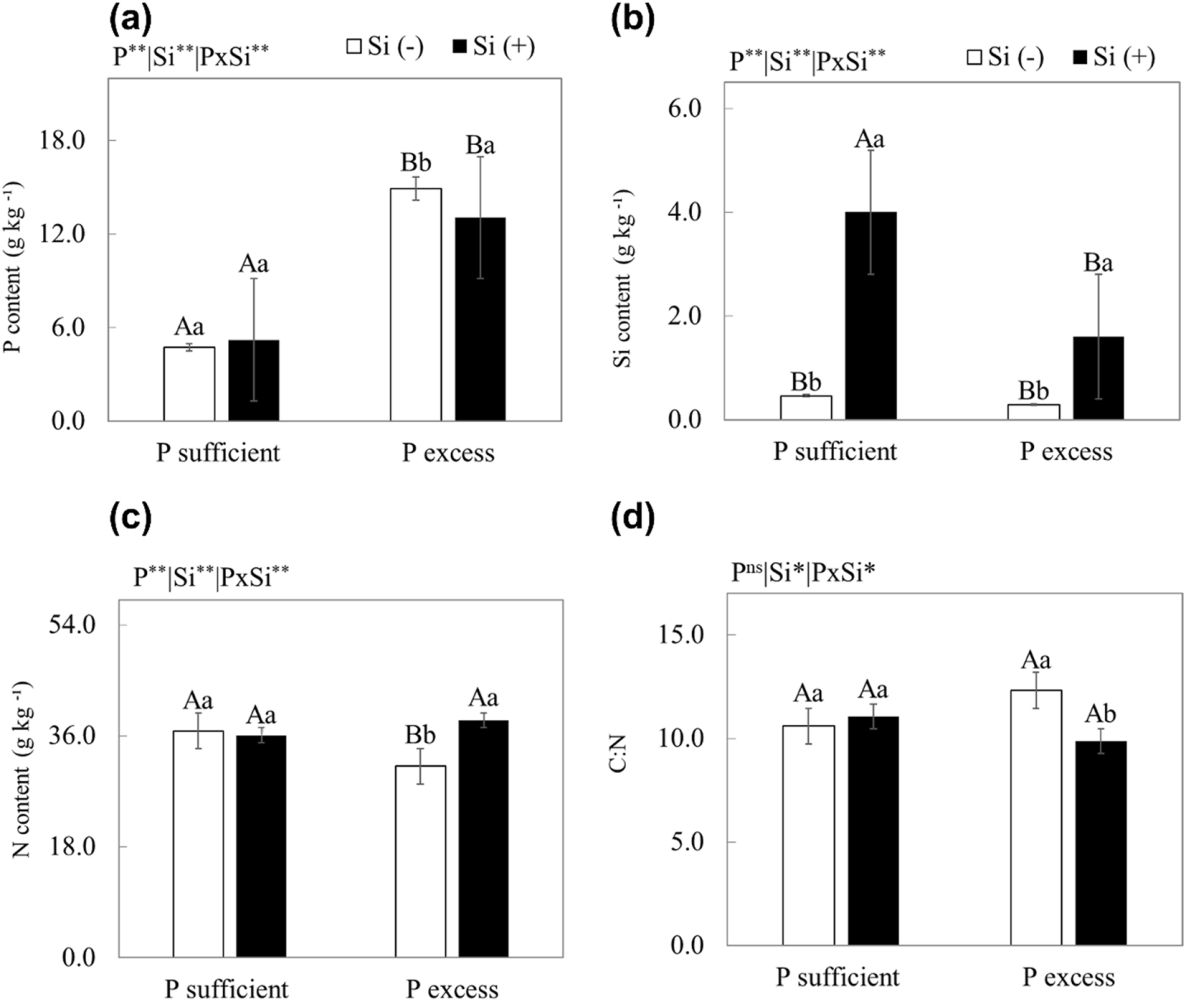
P (**a**), Si (**b**), N (**c**) content and C:N ratio (**d**) of scarlet eggplant cultivated in nutrient solution with adequate and excess P in the absence (− Si) and the presence of silicon (+ Si). Different uppercase letters mean between conditions of P (adequate or excess) within the same Si level (*p* < 0.05) and lowercase letters indicate differences between treatments in the absence and presence of Si within the same level of P (*p* < 0.05). The F test was applied: *(*p* ≤ 0.05) and **(*p* ≤ 0.01). Both were determined by Tukey's test at 5% probability *(*p* < 0.05) e ** (*p* < 0.01). The bars represent the mean standard error. n = 6.

Plants grown with excess P in the absence or presence of Si in the nutrient solution increased the P content in the plant (*p* < 0.01) (Fig. 2a). Only in plants cultivated with an excess of P in the nutrient solution did the addition of Si, in relation to the absence of Si, result in a decrease in P content (*p* < 0.01) (Fig. 2a).

The N content decreased (*p* < 0.01) when plants were cultivated in a nutrient solution with excess P compared to adequate P, especially in the absence of Si (Fig. 2c). However, supplying Si only increased the N content (*p* < 0.01) in plants cultivated with excess P (Fig. 2c).

Plants cultivated with nutrient solution with excess P in relation to adequate P in the absence of Si promoted an increase in the C:N ratio. However, under P excess, Si application decreased the C:N ratio (*p* < 0.05) (Fig. 2d).

**Figure 3:**
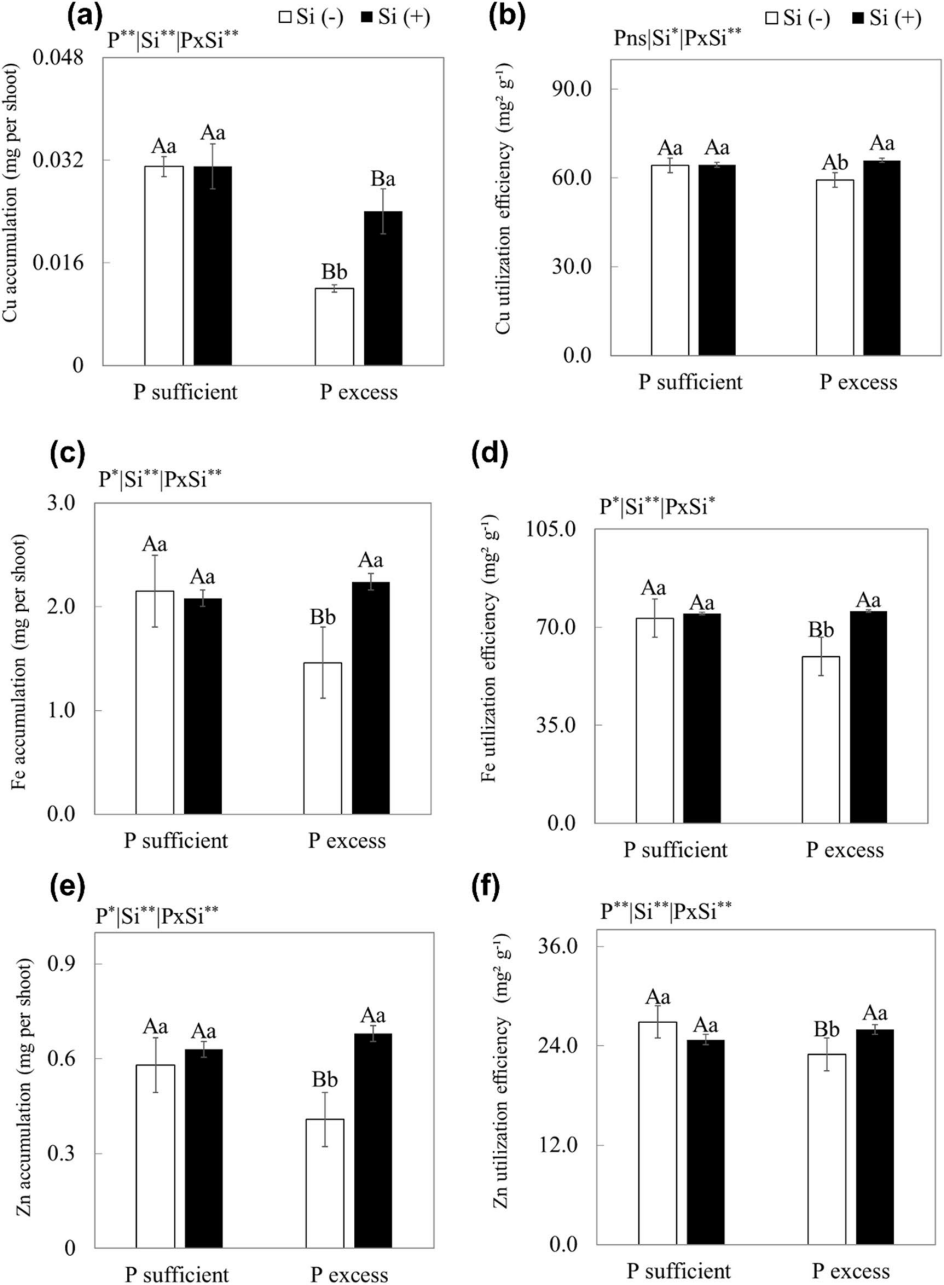
Cu accumulation (**a**), Cu use efficiency (**b**), Fe accumulation (**c**), Fe use efficiency (**d**), Zn accumulation (**e**), Zn use efficiency (**f**) in scarlet eggplant plants grown in nutrient solution with adequate and excess P in the absence (-Si) and presence of silicon (Si +). Different uppercase letters mean between conditions of P (adequate or excess) within the same Si level (*p* < 0.05) and lowercase letters indicate differences between treatments in the absence and presence of Si within the same level of P (*p* < 0.05). The F test was applied: *(*p* ≤ 0.05) and **(*p* ≤ 0.01). Both were determined by Tukey's test at 5% probability *(*p* < 0.05) e ** (*p* < 0.01). The bars represent the mean standard error. n = 6.

Plants grown in nutrient solution with excess P, compared to plants with adequate P, decreased the Cu, Fe, and Zn accumulation by 61%, 32%, and 29%, respectively (*p* < 0.05). The use of excess P in the nutrient solution with the application of Si in relation to plants with adequate P, in the absence or presence of Si, promoted an increase in the accumulations of Cu, Fe, and Zn (*p* < 0.01) (Fig. 3a, c, e).

The excess P in relation to adequate P in the presence and absence of Si resulted in a decrease in the use efficiency of only Fe and Zn (*p* < 0.01). The excess P in the presence of Si, in relation to the absence of Si, increased the use efficiency of Cu, Fe, and Zn (*p* < 0.01) (Fig. 3b, d, f).

**Figure 4:**
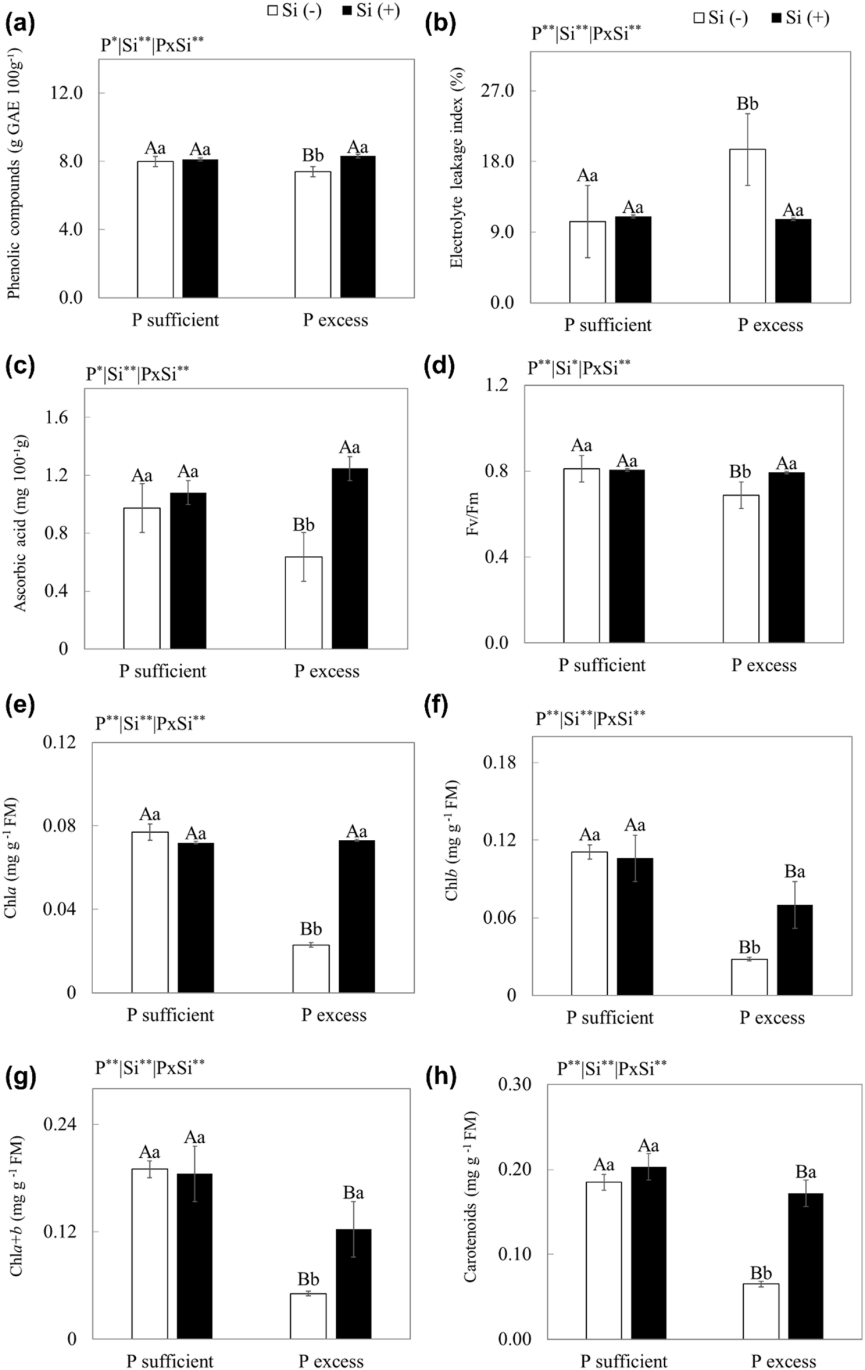
Total phenol content (**a**), electrolyte leakage index (**b**), ascorbic acid (**c**) photosystem II quantum efficiency (d, Fv/Fm), chlorophyll a (Chla, e), chlorophyll b (Chlb, f), total chlorophyll (Chla + b, g), and carotenoids (h) of scarlet eggplant plants grown in nutrient solution with adequate and excess P in the absence (− Si) and presence of silicon (+ Si). Different uppercase letters mean between conditions of P (adequate or excess) within the same Si level (*p* < 0.05) and lowercase letters indicate differences between treatments in the absence and presence of Si within the same level of P (*p* < 0.05). The F test was applied: *(*p* ≤ 0.05) and **(*p* ≤ 0.01). Both were determined by Tukey's test at 5% probability *(*p* < 0.05) e ** (*p* < 0.01). The bars represent the mean standard error. n = 6.

The use of a nutritive solution with excess P in relation to adequate P, in the absence and the presence of Si, resulted in an increase in extravasation of electrolytes and a decrease in the contents of total phenols, ascorbic acid, quantum efficiency of the photosystem II, total chlorophyll, and carotenoids (*p* < 0.01) (Fig. 4).

However, the use of Si in the nutrient solution with excess P compared to excess P without Si decreased electrolyte leakage by 45% and increased levels of total phenols, ascorbic acid, total chlorophyll, and carotenoids by 13%, 50%, 58% and 59%, respectively (*p* < 0.01) (Fig. 4).

**Figure 5:**
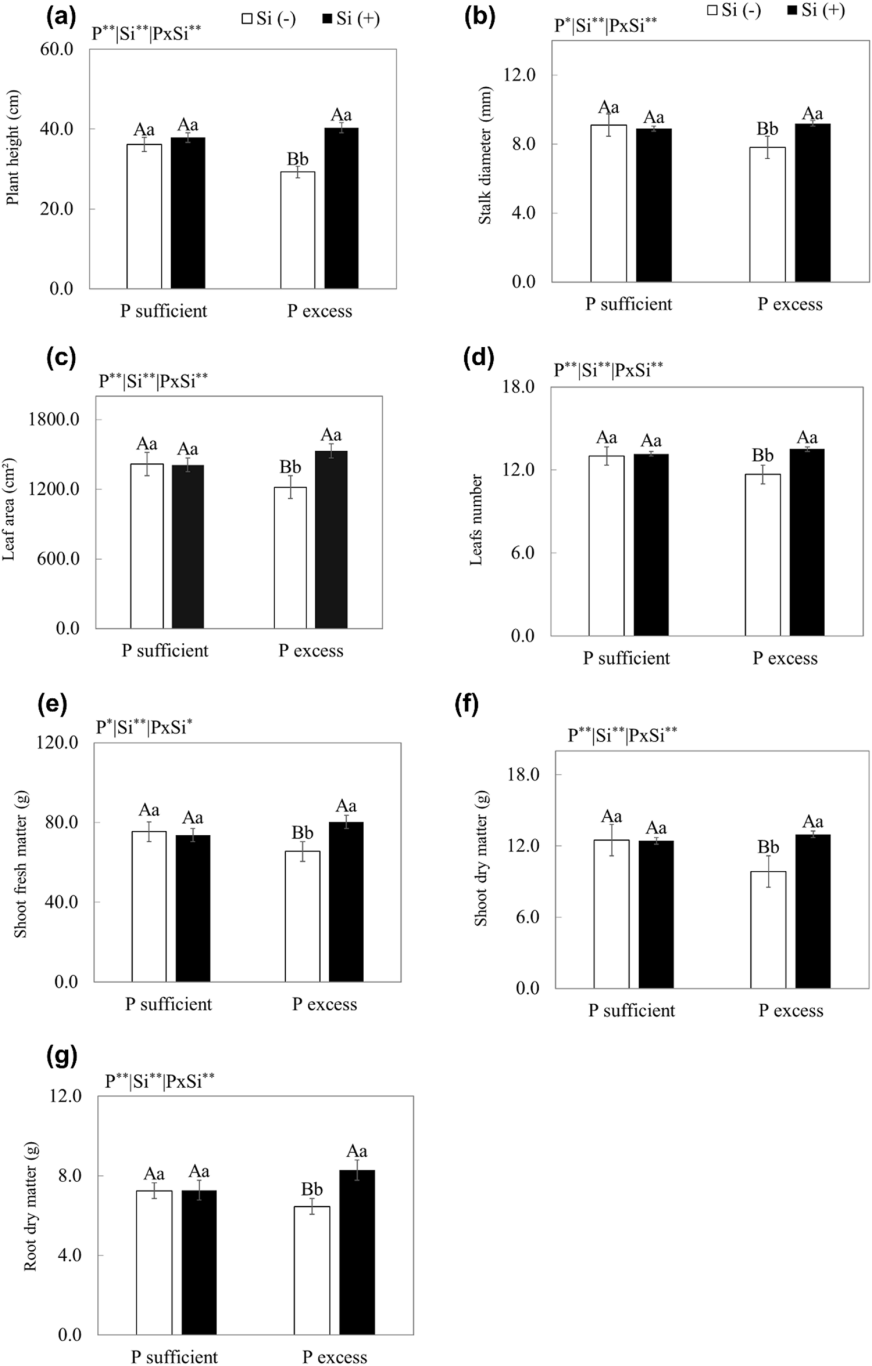
Leaf area (**a**), number of leaves (**b**), plant height (**c**), stem diameter (**d**), fresh mass (**e**), shoot dry mass (**f**), and root dry mass (**g**) of scarlet eggplant grown in nutrient solution with adequate and excess P in the absence (− Si) and presence of silicon (+ Si). Different uppercase letters mean between conditions of P (adequate or excess) within the same Si level (*p* < 0.05) and lowercase letters indicate differences between treatments in the absence and presence of Si within the same level of P (*p* < 0.05). The F test was applied: *(*p* ≤ 0.05) and **(*p* ≤ 0.01). Both were determined by Tukey's test at 5% probability *(*p* < 0.05) e ** (*p* < 0.01). The bars represent the mean standard error. n = 6.

Plants grown with excess P in the nutrient solution, compared to adequate P, in the presence and absence of Si, showed a decrease in height, stem diameter, number of leaves, leaf area, fresh mass, and dry mass of shoots and roots (*p* < 0.05). The supply of Si in relation to Si absence with excess P promoted an increase in the growth variables studied, reaching an increase of 23% and 25% for shoot and root dry mass, respectively (*p* < 0.01) (Fig. 5).

## Discussion

### Effects of P toxicity without Si application

**Figure 6:**
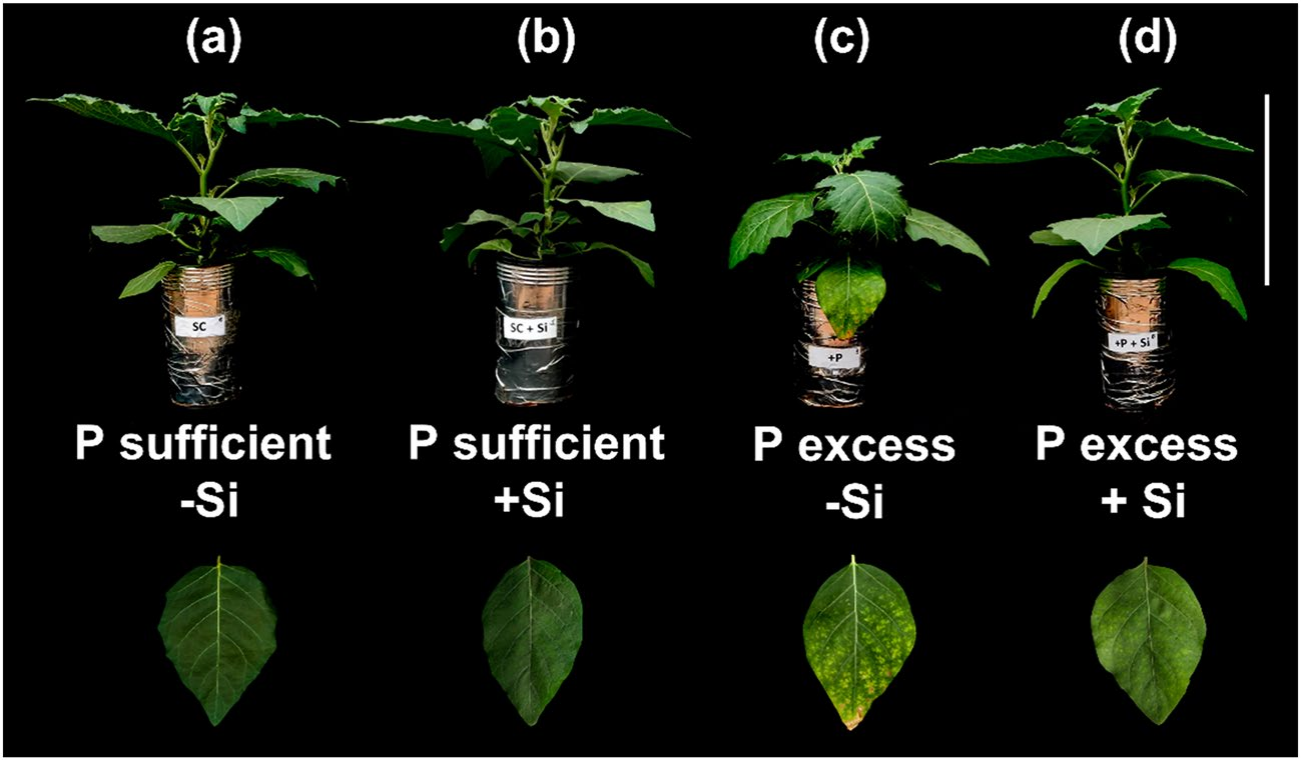
Scarlet eggplant plants grown in nutrient solution with adequate P in the absence of Si (− Si) (**a**) and the presence of Si (+ Si) (**b**) and with excess P in the absence of Si (c) and in presence of Si (d). Bar scale: 35 cm.

Despite the few studies analysing the supply of P in excessive amounts to plants, it is known that plants have developed mechanisms of adaptation to low and high concentrations of P^14^. The toxicity by this element occurs when the concentration of P exceeds 10 g kg^−1^ (or 1%) in dry mass^14,51^. This occurred here in plants cultivated with excess P in the absence of Si, as it reached a content of P in shoots equal to 14.9 g kg^−1^ (Fig. 2a). Even the P levels in plants from P excess treatments had three times the P levels of plants grown with adequate P. This indicates that the treatment application was sufficient to induce toxicity to plants (Fig. 2b), a fact noted visually in scarlet eggplants (Fig. 6).

Therefore, our research is the first to report on the biological effects of excess P on scarlet eggplants. This is important because regions with an intensive cultivation use high doses of P, causing damage to plants^52^. Nevertheless, these disorders are poorly documented.

Thus, in the present research, the toxicity of P initially caused a decrease in the levels of N (Fig. 2c), which may have been due to the decrease in the absorption of the macronutrient since, in the literature, such decrease was reported for the nutrients of plants subjected to P toxicity^53^. However, it was not enough to cause loss of C:N homeostasis in plants^54^.

However, the most important nutritional damage of P toxicity in relation to plants with an adequate P content occurred due to the decrease in the accumulation of micronutrients (Cu, Fe and Zn) in the plant in the absence of Si (Fig. 3a, c , e). This has also been reported for other species^55^. Precipitation of P and metallic micronutrients in the plant root may occur^13^. It is possible that the effects of excess P on the reduction of this micronutrient may vary according to the element since, in the case of Zn, the phosphate anion may have reduced the solubility and availability of Zn in solution^56^; for Mn, there was direct relation shown in the process of absorption of this element by the plant^57^.

This situation probably reduced the transport of these nutrients to leaves, decreasing the activity of these elements in plant metabolism. P is involved in the activity of enzymes and electron transport, decreasing the synthesis of different vital organic compounds in physiological processes in plants^13^. As a result, P toxicity resulted in a decrease in the use efficiency of Cu, Fe and Zn in plant metabolism, causing a decrease in plant dry mass production (Figs. 3 and 4).

Another damage caused by P toxicity to plants is oxidative stress^51^. There was evidence of an increase in cell electrolyte leakage (Fig. 4b). This is possibly due to the increased production of reactive oxygen species, which causes oxidation of lipids in the plant’s cell membrane and damage and increases leakage of cell contents into the space between cells^58^. This occurred because P toxicity left the plant's antioxidant defence system vulnerable due to the significant decrease in the contents of antioxidant compounds, phenols, ascorbic acid, and carotenoids (Fig. 4a, c, h). Consequently, there is no elimination of reactive oxygen species, and the tendency would then be an increase in oxidative stress. This oxidative damage caused by P toxicity reflected in the decrease in the quantum efficiency of the photosystem II (Fig. 4d), a fact other authors reported^59^. It impairs the efficiency of photosynthesis; it is then an indication of stress in plants. This stress indicates that P toxicity causes deleterious effects on chloroplasts. Chlorophyll fluorescence indicates the level of excitation of the energy that drives photosynthesis. It also provides subsidies to estimate the inhibition or damage to the electron transfer process of the PSII. Furthermore, excess P in the cytoplasm may also decrease the activation of enzymes such as fructose-1,6-bisphosphatase, ribulose-5-phosphate kinase, and the concentrations of phosphorylated metabolites^60^, which are important to plant metabolism.

Therefore, the results indicating biological damage by P toxicity explain the damage to growth variables and consequently to dry mass production (Fig. 5) as plant metabolism was limited^61^. This led to visual symptoms of toxicity in the plant. This is the first report of P toxicity symptoms in scarlet eggplant plants grown with excess P without Si, a toxicity verified by chlorotic and reddish spots on older leaves that evolved to necrotic spots on the leaf blade (Fig. 6d).

In conditions of high concentration of P in the cell (excess of P), the transport of P to the vacuole can be mediated by a low-affinity transporter with high Km values^62^. However, due to this limitation of the transporter system, this plant strategy may not be sufficient to completely prevent the occurrence of P toxicity.

We found that the overall damage caused by P toxicity to scarlet eggplant plants involves mechanisms related to nutritional aspects that decrease the nutritional efficiency of micronutrients associated with oxidative stress and increase cell electrolyte leakage due to decreases in antioxidant compounds. Moreover, there was loss of the plant's photosynthetic efficiency, compromising scarlet eggplant growth. Therefore, the results obtained here indicate that the species studied is sensitive to P toxicity, and consequently there is a need for studies proposing sustainable strategies to mitigate this damage to the plant. A promising option is the use of Si, but it still needs further investigation.

### Effects of Si on plants with and without P toxicity

The importance of Si in mitigating stresses of different natures, such as biotic and abiotic stresses, is well known ^63^. However, in scarlet eggplant plants, there are no studies evaluating plant response to the supply of Si, especially attenuating the stress caused by P toxicity. Thus, studies in this line of research should advance towards a better understanding of whether Si is effective in mitigating this stress. If so, it is important to know which its mechanisms are.

In plants under P toxicity, the supply of Si, in relation to its absence, managed to reduce the P content in leaves (Fig. 2a). This is an important strategy to attenuate the damage of excess P to the plant. Although there are a few recent studies on the use of Si to decrease P contents, it is possible that Si may decrease the activity of P transporters in the plant ^64^, and that Si may form an apoplastic barrier that prevents the absorption of excess P ^65^. The decrease in P content induced by Si in plants with P toxicity caused an improvement in stoichiometric homeostasis by decreasing the C:N ratio as it increased the N content (Fig. 2c) and decreased the C content. In other species (*Brassica napus* L.), the supply of Si also increased the N content due to an increased absorption of the N ^51^. It is possible that Si in stressed plants favours the stoichiometric homeostasis of both nutrients (C and N), optimizing their vital biological functions for an optimal plant metabolism^13^. There are indications that Si, when in structural compounds of the cell wall replacing C in organic chains, saves energy for the plant since the energy cost for including Si into organic compounds is lower compared to that of C ^66^. This is an important fact for a plant suffering a stress that demands a greater pressure for energy to be used in metabolism homeostasis.

Another important Si mechanism to mitigate P toxicity is that it increases the accumulation and the use efficiency of Zn, Cu and Fe (Fig. 3) with the application of Si compared to absence of Si. A well-studied nutritional antagonist could be P and Zn. Si attenuates this nutritional disorder by decreasing the P content and, at the same time, increasing the Zn content when P is in excess^67^. It should be noted that this increase in the use efficiency of the micronutrients studied here promoted by Si in plants with P toxicity is possibly due to its effect on a greater translocation and redistribution of these elements in plant tissues^68^.

Si affects the expression of genes involved in the biosynthesis of mobilizing compounds such as citrate, which is a Fe chelator, for example in the xylem, increasing the translocation of the metallic micronutrient to the shoot^69^. A similar effect of Si is redistribution. Si increases the expression of the NAS1 gene, which is responsible for the biosynthesis of NA (nicotianamine amino acid). Therefore, Si increases NA accumulation, which in turn increases micronutrient chelation and the loading of Si in the phloem, thus favoring phloem mobility^70^.

The effect of Si benefiting Cu, Fe and Zn nutrition in scarlet eggplant plants with P toxicity has two important consequences that help to understand better this mitigation. Si, by increasing the absorption and accumulation of these micronutrients, should directly favour several well-known biological functions of these elements. For example, Zn acts in enzymatic systems involved in the synthesis of the amino acid tryptophan, responsible for the synthesis of AIA, which stimulates plant growth and other functions, and Cu and Fe, which show an enzymatic action in different metabolic routes and in the transport of electrons, among other functions^13^. The second development is linked to the first, that is the attenuation of P toxicity, as these micronutrients perform specific functions that favour the plant's antioxidant defence system. This is because Cu and Zn^71^ are part of the structure of important enzymes (superoxide dismutase, ascorbate peroxidase, catalase) that are responsible for the decomposition reaction of reactive oxygen species into water and oxygen.

This benefit of Si in reducing oxidative stress in scarlet eggplant plants is proven by the increase in antioxidant compounds in plants. We showed that the supply of Si, in relation to the absence of Si, in plants with P toxicity resulted in an increase in the production of total phenols (Fig. 4a), ascorbic acid (Fig. 4c), and carotenoids (Fig. 4h). Although this effect of Si on the increase of the antioxidant compounds phenols and ascorbic acid in plants under stress condition^71,72^ has been reported for other species, in scarlet eggplant plants this effect was as of yet unknown.

Another aspect that proves the antioxidant effect of Si on plants is that the increase in these antioxidant compounds was enough to reduce the leakage of cellular electrolytes (Fig. 4b). As previously mentioned, Si reinforces the plant's antioxidant defence system^73^ and decreases the degradation of cell membranes^74^.

This antioxidant effect promoted by Si preserved photosynthetic pigments as it increased the chlorophyll a, b, and a + b contents in plants with P toxicity that received Si (Fig. 4g). In addition, Si, by increasing the N content, as seen above, contributed to an increase in chlorophyll synthesis, as N constitutes its structure^75^.

These benefits of Si, in addition to the attenuation of P toxicity, favoured the growth of scarlet eggplants as the application of Si, in relation to its absence, increased plant height, stem diameter, number of leaves, and leaf area (Fig. 5a, b, c, d), and consequently increased the production of dry mass (Fig. 5f).

This study shows for the first time in scarlet eggplant plants that the use of Si can be a robust strategy to mitigate P toxicity. This finding has global implications since cultivation with unbalanced P fertilization occurs in several regions of intensive cultivation of vegetables, especially close to large cities. The present research also presents the protection mechanisms Si promotes to mitigate P toxicity in scarlet eggplant. It shows the association of nutritional improvements from the decrease in P content to the increase in the nutritional efficiency of Fe, Zn and Cu. At the same time, it markedly affects the strengthening of antioxidant defence induced by the synthesis of antioxidant compounds.

The present research also studied the responses of scarlet eggplant plants to the application of Si without stress or with sufficient P. Si, because it is a beneficial element, is not essential for crops. However, responses to Si are common in different crops, especially in plants under stress, a fact that occurred here with eggplant plants under P toxicity, as discussed earlier. However, studies on stress-free cultivation are scarce, with no reports on scarlet eggplant. This research shows that in scarlet eggplant plants without stress, that is, with sufficient P, the inclusion of Si in the nutrient solution, in relation to its absence, does not affect any variable analysed. This may occur because Si plays a greater role in plants that suffer some type of stress^76^. Therefore, in areas where scarlet eggplant is cultivated without P imbalance, the supply of Si is not recommended because it does not promote any benefit to plant growth due to the excellent metabolism of the plant.

Future studies should advance on metabolomics as it is possible that these physiological and clear biochemical improvements promoted by Si may result from the ability of the beneficial element to modify the gene expression of the plant.

## Conclusions

Scarlet eggplant culture was sensitive to excess P in the culture medium. The decrease in growth of this plant occurs because of biological damage, nutritional damage, and oxidative stress. We show that P toxicity in scarlet eggplant plants is modulated by the supply of Si via nutrient solution at a concentration of 2 mmol L^−1^ because it decreases P absorption, improves C:N homeostasis, and increases the use efficiency of Fe, Cu, and Mn. At the same time, Si reduces oxidative stress and favours photosynthetic efficiency and plant growth.

These findings allow elucidating the potential of using Si as an effective and sustainable strategy to reverse the damage caused by P toxicity in fertigated scarlet eggplant plants.

## Data Availability

The datasets generated and/or analyzed during the current study are available from the corresponding author on reasonable request.
